# Internal tissue references for ^18^Fluorodeoxyglucose vascular inflammation imaging: Implications for cardiovascular risk stratification and clinical trials

**DOI:** 10.1371/journal.pone.0187995

**Published:** 2017-11-13

**Authors:** Mark A. Ahlman, Davis M. Vigneault, Veit Sandfort, Roberto Maass-Moreno, Jenny Dave, Ahmed Sadek, Marissa B. Mallek, Mariana A. F. Selwaness, Peter Herscovitch, Nehal N. Mehta, David A. Bluemke

**Affiliations:** 1 Radiology and Imaging Sciences, National Institutes of Health, Bethesda, MD, United States of America; 2 Institute of Biomedical Engineering, Department of Engineering, University of Oxford, Oxford, United Kingdom; 3 Sackler School of Graduate Biomedical Sciences, Tufts University School of Medicine, Boston, MA, United States of America; 4 National Heart, Lung, and Blood Institute, National Institutes of Health, Bethesda, MD, United States of America; 5 PET Research Department, National Institutes of Health, Bethesda, MD, United States of America; Biomedical Research Foundation, UNITED STATES

## Abstract

**Introduction:**

^18^Fluorodeoxyglucose (FDG) positron emission tomography (PET) uptake in the artery wall correlates with active inflammation. However, in part due to the low spatial resolution of PET, variation in the apparent arterial wall signal may be influenced by variation in blood FDG activity that cannot be fully corrected for using typical normalization strategies. The purpose of this study was to evaluate the ability of the current common methods to normalize for blood activity and to investigate alternative methods for more accurate quantification of vascular inflammation.

**Materials and methods:**

The relationship between maximum FDG aorta wall activity and mean blood activity was evaluated in 37 prospectively enrolled subjects aged 55 years or more, treated for hyperlipidemia. Target maximum aorta standardized uptake value (SUV) and mean background reference tissue activity (blood, spleen, liver) were recorded. Target-to-background ratios (TBR) and arterial maximum activity minus blood activity were calculated. Multivariable regression was conducted, predicting uptake values based on variation in background reference and target tissue FDG uptake; adjusting for gender, age, lean body mass (LBM), blood glucose, blood pool activity, and glomerular filtration rate (GFR), where appropriate.

**Results:**

Blood pool activity was positively associated with maximum artery wall SUV (β = 5.61, *P*<0.0001) as well as mean liver (β = 6.23, *P*<0.0001) and spleen SUV (β = 5.20, *P*<0.0001). Artery wall activity divided by blood activity (TBR_Blood_) or subtraction of blood activity did not remove the statistically significant relationship to blood activity. Blood pool activity was not related to TBR_liver_ and TBR_spleen_ (β = −0.36, *P* = NS and β = −0.58, *P* = NS, respectively)

**Conclusions:**

In otherwise healthy individuals treated for hyperlipidemia, blood FDG activity is associated with artery wall activity. However, variation in blood activity may mask artery wall signal reflective of inflammation, which requires normalization. Blood-based TBR and subtraction do not sufficiently adjust for blood activity. Warranting further investigation, background reference tissues with cellular uptake such as the liver and spleen may better adjust for variation in blood activity to improve assessment of vascular activity.

## Introduction

Atherosclerosis is the leading cause of mortality in the western world and anatomic imaging is gaining a central role in its evaluation [1, 2]. Recent evidence suggests that positron emission tomography (PET) vascular imaging with agents such as ^18^Fluorodeoxyglucose (FDG) may provide additional diagnostic and prognostic information about arterial inflammation over anatomic imaging alone [3] by disclosing imaging biomarkers of pre-clinical cardiovascular risk as well as by showing rapid changes in vascular uptake following treatment [4]. These potential advantages ushered the use of FDG vascular imaging in clinical treatment trials [5].

A PET imaging biomarker for vascular inflammation would have great value if it adds additional information on disease status independent of technical factors or cardiovascular risk factors that can otherwise be easily corrected for or measured. The optimal methodology for quantifying vascular activity remains in question [6–9]. Some of the challenges have been well described, such as differences in hardware and image reconstruction [8], as well as variation of subject blood glucose, uptake time, and FDG dose [10]. However, some intrinsic physical and pharmacodynamic PET imaging elements may be indirectly or directly related to components of cardiovascular risk, and therefore confound its use for risk stratification if used without appropriate normalization.

**Fig 1:**
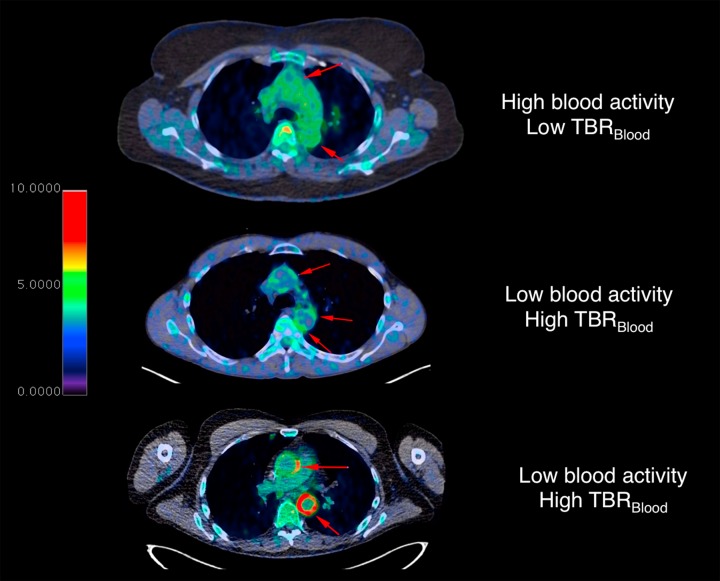
Examples of variation in blood activity and aorta wall FDG uptake. PET (color) and CT (black/white) axial fusion images just below the aortic arch of three subjects, using identical windowing and leveling of PET SUV according to the look up table (left). Red arrows point to regions where aorta wall activity is expected. The top subject has low TBR_Blood_ with high levels of blood activity in the aorta lumen (green). The middle subject has lower blood activity in the lumen of the aorta, which allows visual contrast of the aorta wall, therefore higher TBR_Blood_. It is not clear if the difference in TBR_Blood_ between the top two subjects is related to differences in inflammation or due to differences in blood activity. However, compared to a subject with active large vessel vasculitis (bottom), there is clearly abnormal aorta wall activity that can be distinguished from blood activity.

A key difference between glucose and FDG is that FDG undergoes renal elimination from the body at a rate moderated by glomerular filtration rate (GFR) [9, 11–13]. However, renal dysfunction is an a independent risk factor for cardiovascular disease [14–16] and GFR is closely linked to age [17]. The issue is further complicated knowing that GFR is potentially modified by statin treatment [18]. These associations are important in vascular imaging with PET tracers, in part due to the immediate juxtaposition of the target vessel wall to blood pool activity, which cannot be easily spatially discriminated in PET imaging [9]. However, the issue expands beyond simply correcting for blood activity at a specific time point. Variation of the concentration of FDG available to a target artery wall lesion during the entire uptake phase is also a driving factor, which is moderated within a closed system by FDG elimination as well as competition for its uptake by other cells in the body. Early validation and current methodology relies on the use of blood pool activity as a background reference for the normalization variable for target vessel wall activity to compute a target-to-background ratio (TBR or TBR_Blood_) [19, 20]. This method is reproducible [21] and has been the primary method used in clinical trials [20, 22]. The purpose of such normalization is to define a quantitative measure desensitized to variations in the amount of available tracer (i.e. activity in the blood). However this compensation may be insufficient [6, 9]. As shown in Fig 1, the greater the activity in the blood, the lower the TBR but also the higher the spill-in from the blood contributing to the measured vessel wall activity (which is itself subject to partial volume effects). Thus, alternate internal background tissue references other than blood itself may be desired. For instance, tissues easy to measure such as liver may be robust background reference tissue despite expected changes in activity related to therapy in oncology, is resistant to variability in uptake time, and is used clinically [23]. The liver is used for normalization in FDG-PET for vasculitis [24] and oncology [23], but has not been systematically evaluated for the same purpose for lesions expected to have comparably low activity, such as the atherosclerotic plaque.

In a relatively homogeneous population with hyperlipidemia, the first goal of this study was to evaluate if the use of blood-based TBR (TBR_Blood_, as defined above) or if the simple subtraction of blood activity from target artery wall activity removes any statistically significant multivariate relationship between these variables and blood pool activity. The second goal was to perform the same in vivo evaluation using tissues such as the liver and spleen instead of blood activity to calculate TBR. The third goal was to evaluate plausible factors that may modify blood or vessel wall FDG activity, as applied to the use of FDG PET for vascular imaging.

## Materials and methods

### Human subjects

The institutional review board at the National Institutes of Health approved the study (NCT01212900). All procedures performed in studies involving human participants were in accordance with the declaration of Helsinki. Written informed consent was obtained from all participants. 37 subjects with hyperlipidemia over the age of 55 were prospectively enrolled. Imaging included two FDG PET-CT scans at baseline and 6 months. Subjects with symptoms characteristic of angina or heart failure were excluded. A prior or current history of hypertension, smoking, or diabetes was noted. Self-reported race/ethnicity, gender, and baseline blood pressure, as well as Framingham risk score were recorded. The serum creatinine level closest in time to imaging was used to estimate GFR [17].

### Scanning procedures

Subject preparation included fasting for at least six hours with instructions to consume a meal low or absent of carbohydrates the evening before imaging the following morning. A blood glucose measurement of greater than 200 mg/dl precluded FDG injection. Per the convention for vessel wall imaging [7], more than 2 hours uptake time was used to assure that maximum activity measurement over the aorta was due to artery wall activity and less so from excessive blood activity in the lumen. FDG dosing was based on lean body mass (LBM) (5.2 MBq/kg, 0.14 mCi/kg), and imaging proceeded with a Biograph mCT (Siemens Medical Solutions, Erlangen, Germany), a 128 CT detector row PET-CT. PET images were obtained from the upper thighs to above the orbits in a caudocranial direction using 3 min/bed (7 beds). Reconstruction included CT attenuation correction (120 kVp, 56 effective mAs, 1.5mm thickness, 512x512 matrix) without adaptive tube current modulation. PET image reconstruction (24 iterations, 3 subsets, with point spread function correction, without post-reconstruction filtering) produced a 256 x 256 matrix volume with a final voxel size of 3.18mm X 3.18mm X 1.5 mm after applying 1.2 zoom.

### Image analysis

Image segmentation and measurement was performed with Osirix™ version 5.8.5 (Pixmeo SARL, Geneva, Switzerland). Whole-vessel regions of interest were carefully placed on each slice (approximately 300 slices) over the thoracic and abdominal descending aorta to obtain the mean of maximum voxel intensities from each slice; with care to avoid non-vascular activity. The inferior and superior vena cava and the visualized portion of the left jugular vein were segmented to estimate mean blood pool activity. A volume of interest was obtained within the center of the right lobe of the liver to determine mean liver activity, with exclusion of the inferior vena cava. Examples of segmentation are further detailed in supplemental S1 and S2 Figs. Standardized uptake values (SUV) normalized to LBM were calculated using the acquisition start time as the adaptive slice-position imaging time used for decay correction.

Arterial target to background ratios (TBR) were calculated using blood (TBR_Blood_), liver (TBR_Liver_), and spleen (TBR_Spleen_) as background reference tissues. Subtraction measurements were obtained by subtraction of mean blood activity from mean of maximum arterial wall SUV.

### Statistical analysis

Unless otherwise specified, continuous values are reported as median and inter-quartile range (IQR). To weigh the factors that potentially modify blood activity, multivariate analysis was utilized. With blood activity as the dependent variable, GFR, gender, age, LBM, and glucose were used as covariates. To remove potential interaction, GFR was removed as a covariate from subsequent analysis in favor of the placement of blood activity itself into the model as a covariate. Thus, to evaluate potential alternate background reference tissues, the mean SUV of the liver and spleen were subsequently placed as dependent variables with covariates of blood SUV, gender, age, LBM, and glucose. These same covariates were then used to investigate the various methods of arterial activity as dependent variables (Aorta SUV, TBR_Blood_, TBR_Liver_, TBR_Spleen_, and blood subtraction).

Care was applied to standardize uptake time. Thus, it was not included in the models due to sufficiently low variability (<5% coefficient of variation). The final model included both baseline and 6-month time points (total scans, n = 74) using the generalized estimating equation to minimize within-subject correlation errors using R version 3.0.2 (The R Foundation for Statistical Computing, Vienna, Austria). The mean, standard deviation (SD), and coefficients of variation (COV) of image measurement variables at the baseline examination are reported in the supplemental material (S1 Table), and were used to calculate normalized beta coefficients. *P* values <0.05 were considered statistically significant.

## Results

Subject demographics and imaging characteristics are shown in Table 1. The median age was 63.0 (59.0, 67.0) years and 41% of subjects were female. The median body mass index was 27.1 (25.2, 29.8) and the median GFR proximal to the time of imaging was 80.5 (68.3, 93.7) ml/min/1.73m^2^. Median glucose at the time of imaging was 89.0 (84.0, 97.0) mg/dl, indicating a predominantly euglycemic state for the population at the time of imaging.

**Table 1:** Subject characteristics (n = 37) and imaging parameters.

Female (%)	15 (41%)
Age (years)	63.0 (59.0, 67.0)
Weight (kg)	81.3 (72.5, 91.1)
Body Mass Index (kg/m^2^)	27.1 (25.2, 29.8)
Lean Body Mass (kg)	56.17 (50.7, 63.6)
Current or past smoker %	22 (59%)
Diabetic %	0 (0%)
Framingham risk %	7.0% (2.0, 12.0)
Ethnicity	
Hispanic	1 (3%)
Black	3 (8%)
White	31 (83%)
Asian	1 (3%)
Other	1 (3%)
Systolic Blood Pressure (mm Hg)	130.0 (121.5, 136.0)
Diastolic Blood Pressure (mm Hg)	73.0 (65.0, 79.5)
Total Cholesterol (mg/dl)	187.0 (157.5, 208.0)
LDL (mg/dl)	100.0 (80.5, 119.5)
Triglycerides (mg/dl)	103.0 (71.5, 139.5)
Glucose (mg/dl)	89.0 (84.0, 97.0)
Uptake time (minutes)	136.0 (133.8, 140.0)
GFR (ml/min/1.73 m^2^)	^†^80.5 (68.3, 93.7)
Dose (MBq)	294.3 (256.9, 330.6)

Data reported as median (IQR), or n (%) unless otherwise specified

^†^Serum creatinine within a median of 0.0 (0.0, 9.5) days of imaging

Shown in Table 2, there were no statistically significant (*P≥*0.05) variables independently associated with blood pool activity. However, when corrected for gender, age, LBM, and glucose, there was a strong positive relationship of blood activity to both liver (β = 6.23, *P*<0.0001) and spleen (β = 5.20, *P*<0.0001) activity.

**Table 2:** Multivariate regression models of confounding variables associated with blood, liver, and spleen activity. Column headers are dependent variables and rows are covariates.

	Blood SUV	Liver SUV	Spleen SUV
Blood SUV	**−**	*6.23	*5.20
GFR (ml/min/1.73m2)	**−**1.27	**−**	**−**
Female	**−**0.37	**−**0.55	-0.48
Age (years)	0.33	**−**0.66	**−**1.97
LBM (kg)	2.07	**−**0.88	**−**0.42
Glucose (mg/dl)	**−**1.11	**−**0.40	0.07

*P<0.0001, Normalized beta coefficients reported. Statistically significant variables in bold.

The first column of Table 3 demonstrates that variation in blood SUV was the predominant factor that was associated with artery wall SUV (β = 5.61, *P*<0.0001). Additionally, female gender had significant negative association with aorta SUV (β = **−**0.77, *P*<0.05). However, as shown in the second column of Table 3, there was a significant negative association (β = **−**2.46, *P*<0.05) between blood activity to TBR_Blood_, which indicates that this traditional marker of vascular inflammation may not likely adequately normalize for variation in blood FDG activity in this population. A similar lack of normalization was seen for the alternative of subtracting the blood SUV from the arterial wall SUV, shown in the third column of Table 3 (β = 3.35, *P*<0.001). There is a significant negative association (β = **−**1.20, *P*<0.05) of female gender with TBR_Blood_ as found with arterial SUV, which is not seen when blood subtraction is used (β = 0.09, *P* = NS). Importantly, when TBR_Liver_ and TBR_Spleen_ were measured (last two columns of Table 3), an independent statistically significant relationship to blood SUV was not shown, suggesting that these non-blood background reference tissues, despite their individual significant associations with blood activity (as shown in Table 2), potentially normalizes for variation in blood activity in this population.

**Table 3:** Multivariate regression models of confounding variables associated with measurement methods of arterial activity. Column headers are dependent variables and rows are covariates.

	Artery Wall SUV	TBR_Blood_	Blood Subtraction	TBR_Liver_	TBR_Spleen_
Blood SUV	****5.61	*−2.46	**3.35	**−**0.36	**−**0.58
Female	*−0.77	*−1.20	0.09	**−**0.42	**−**0.43
Age (years)	**−**1.05	**−**1.64	**−**1.56	**−**0.54	1.29
LBM (kg)	**−**0.81	**−**1.26	1.03	**−**0.06	**−**0.52
Glucose (mg/dl)	**−**0.64	**−**1.02	**−**0.75	**−**0.66	**−**1.13

* *P*<0.05

**P<0.01

***P<0.001

****P<0.0001

Normalized beta coefficients reported. Statistically significant variables in bold.

## Discussion

Compared to the other factors investigated, blood FDG activity carries the strongest association with vessel wall activity. Importantly, blood-based TBR likely does not sufficiently normalize for variation in blood activity, despite being the most commonly employed and recommended technique for this purpose [7]. Moreover, our analysis does not support the claim that that blood subtraction from arterial activity better normalizes for blood activity [6]. However, despite the close relationship of blood activity to liver and spleen activity, we found that the use of TBR_Liver_ and TBR_Spleen_ removes a statistically significant relationship to variation in blood activity in this study population for reasons that require further consideration.

A lack of standardization remains for measurement methods used to evaluate PET vascular activity [7–9, 25]. Specific to its potential use as a quantitative imaging biomarker for cardiovascular risk-stratification, the justification for the expense and effort of PET vascular imaging would be to expand upon known cardiovascular risk factors such as race, gender, and age. However, we observe that the relative influence of variation in blood activity on aorta wall maximum SUV, TBR_Blood_, and blood-subtracted aorta wall SUV greatly exceeds the influence from other known cardiovascular risk factors such as gender and age, which addresses the primary question in this study. This high level of influence of blood activity prompts questions about the causes of variation in FDG blood activity itself and about the extent to which it has impacted its validation and subsequent use for measurement of vascular inflammation. It can be said, however, that the results from this investigation showed no statistically significant relationships of GFR, age, gender, LBM, or glucose activity to blood activity, which requires further discussion.

It is known that renal function moderates FDG elimination from the body and has known relationships to gender, age, and race, which are three required variables for GFR estimation using serum creatinine [17]. However, we were unable to sample a statistically significant relationship between GFR and blood activity, which may be related to the lack of a wide variation in renal function for this population of limited sample size and limited racial diversity. Additionally, it is plausible that the demographic elements (age, gender) used to calculate GFR might have resulted in untoward interactions in a multivariate model, given that these elements were necessary separate components of cardiovascular risk modeling. Although we use a similar multivariate strategy for this purpose as other investigators [10, 25], the complexity of these potential interactions is a limitation of the data at hand or our analysis, requiring further investigation.

We did not find that LBM was significantly related to background reference tissue or aorta wall activity. LBM is an approximation of the expected volume of distribution and in vivo concentration of FDG, which is why it is often preferred for SUV calculation in lieu of body weight [23]. However, similar to the collinearity issues discussed with the demographic elements for the calculation of GFR, the calculation of LBM also requires the input of age and gender. Furthermore, there are well-described relationships of body size to PET image noise [26], whereby larger individuals attenuate a higher fraction of photons leaving the body, resulting in higher image noise. Particularly when sampled over multiple slices over the aorta as typically employed in vascular imaging [7], the higher noise is expected to result in apparent higher maximum SUV measurement for larger individuals. While our dosing strategy based on LBM was originally intended to reduce radiation and normalize for the FDG volume of distribution, it was not intended for, and likely not sufficient for correction for variation in image noise related to attenuation in some patients. Because of these uncertainties and the relationship of obesity to atherosclerosis [27], it is challenging to interpret a lack of statistical significance for LBM. Techniques to harmonize image noise [8] were outside the scope of this work, but are endorsed for future consideration.

Glucose is a competitive inhibitor of FDG. Our subjects were within the acceptable blood glucose range of non-diabetic subjects in a fasting state (<200 mg/ml). Thus, the residual variation in blood glucose at the start of imaging was not significantly associated with any measurement of background reference tissue or artery wall activity. This finding may not imply that blood sugar can be ignored in PET vascular imaging, but rather supports that the limits currently recommended to prepare a patient for PET imaging are adequate [7].

Perhaps in line with its recommended use as an internal background tissue reference for oncology imaging [23], we found that the use of liver for TBR calculation removes a statistically significant multivariate relationship to blood pool activity. Without hard measures of disease activity, we acknowledge that this lack of a relationship to blood activity does not necessarily equate with better diagnostic utility. However, we hypothesize that tissues such as the liver better normalize for blood pool activity over blood activity itself for reasons other than ease of measurement. Firstly, we avoid the assumption that the only goal of normalization for blood activity is to correct for the physical spillover of blood activity in the final image due to the limited spatial resolution of PET (e.g. luminal blood adjacent to artery wall). Second, this study is understandably limited by the fact that the number of potential modifiers of background reference tissue or vascular uptake that we evaluate in this study is unlikely to be comprehensive. A broad representation of known or unknown factors are likely at play (e.g. insulin, adequacy of fasting, hydration status, medications, etc.) during the uptake phase prior to imaging as all cells in the body compete for a finite quantity of FDG introduced to a closed system. However, without the current ability to account for all meaningful variables, we emphasize that this dynamic environment exists for the non-target liver or spleen cells as well as for cells of the target artery wall during uptake. However, some caution against the use of non-blood organs as a background tissue references for this purpose due to concerns that these tissues will have altered activity that confounds a true signal of vascular inflammation. For instance, spleen activity responds to systemic inflammation [7] and liver activity may respond to steatosis [28]. We accept these concerns, but are currently not aware of data validating a claim that systemic inflammation would not also alter blood activity itself, as all cells within a closed system compete for the finite quantity of FDG during uptake. In such a scenario, we might instead expect a negative relationship of high organ activity to low blood activity. Rather, we show the opposite strong positive relationship of blood to liver and spleen activity (β>5, *P*<0.0001) and a similar strong positive relationship to aorta wall activity (β>5, *P*<0.0001), which suggests that variation in blood activity itself (and its normalization) must be further addressed when internal quantitative correlations of organ activity are observed. Indeed, there are recent proposals that FDG activity in organs such as fat, periodontal tissue, synovial tissue, spleen, and bone marrow relate to target arterial FDG activity due to a common state of systemic inflammation [19, 29–31]. As shown in this work, without an appreciation that traditional normalization methods such as SUV and TBR_Blood_ may not be expected to effectively correct for a common dynamic influence of blood in all these tissues, such associations may cause confusion. An argument can be made to use the liver rather than the spleen as a background reference tissue for this purpose due to recent evidence that the spleen activity is related to systemic inflammation [32], its well-described and clinically established use in oncology [23], and its more common use as a background reference in vasculitis imaging [24, 33]. Additionally, serologic or imaging markers are available if liver dysfunction is suspected.

Regardless of the specific background reference tissue used, this work substantiates that quantitative comparisons of PET target arterial activity related to treatment or disease state likely requires evidence in parallel that the measured difference is not also reflected in the background reference tissue used for normalization or by external factors that modify apparent vessel wall activity.

We maintain that the expansion of these core results beyond the relatively homogenous and otherwise healthy population studied to be appropriate to a certain extent due to strong statistical significance and the proposed mechanistic plausibility of the relationships. However, risk-stratification for populations of varied age, race and disease state (e.g. diabetes, renal failure) is of interest for the identification of patients that would benefit from earlier or more intense treatment to forestall end-stage vascular damage. We propose that further investigation in these lines may benefit from an appreciation of the key confounding normalization issues described herein.

## Conclusions

Blood pool FDG activity has a high influence on vascular wall activity, which is not corrected for by the use of blood-based TBR or subtraction. With supporting data, a rationale is proposed for the use of non-blood background reference tissues such as the liver for normalization, which warrants further investigation. However, owing to the complex intrinsic collinear relationships of age, gender, body size and blood FDG activity in PET vascular imaging, these factors require special attention for cardiovascular risk-stratification. The results also have implications for treatment-related evaluation and for further study of the cost-benefit of this emerging application of PET imaging.

## Supporting information

S1 Fig Aorta wall and blood segmentation. (TIF)

S2 Fig Segmentation of liver and spleen. (TIF)

S1 File Raw quantitative data. (ZIP)

S1 Table Measurement characteristics. (DOCX)

## Abbreviations

COV: Coefficient of Variation
CT: Computed Tomography
FDG: ^18^Fluorodeoxyglucose
GFR: Glomerular Filtration Rate
IQR: Inter-Quartile Range
LBM: Lean Body Mass
PET: Positron Emission Tomography
SD: Standard Deviation
SUV: Standardized Uptake Value
TBR: Target-to-Background Ratio
