# Supplementary figures and images for: Internal tissue references for 18Fluorodeoxyglucose vascular inflammation imaging: Implications for cardiovascular risk stratification and clinical trials
Source: PLoS One. 2017 Nov 13;12(11):e0187995. doi: 10.1371/journal.pone.0187995 (PMC5683610; doi:10.1371/journal.pone.0187995)

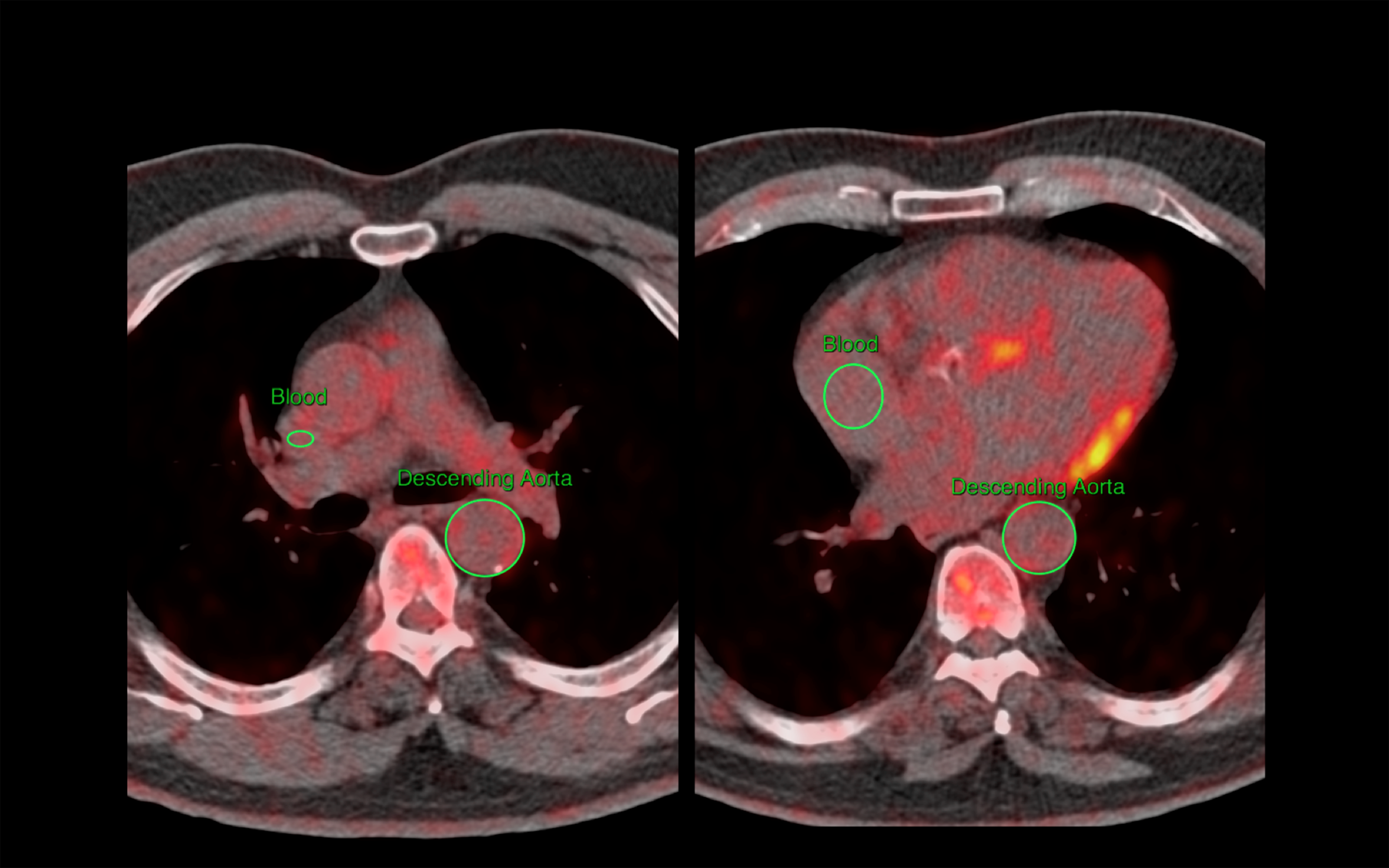

Supplement: S1 Fig — (TIF) [file pone.0187995.s001.tif]

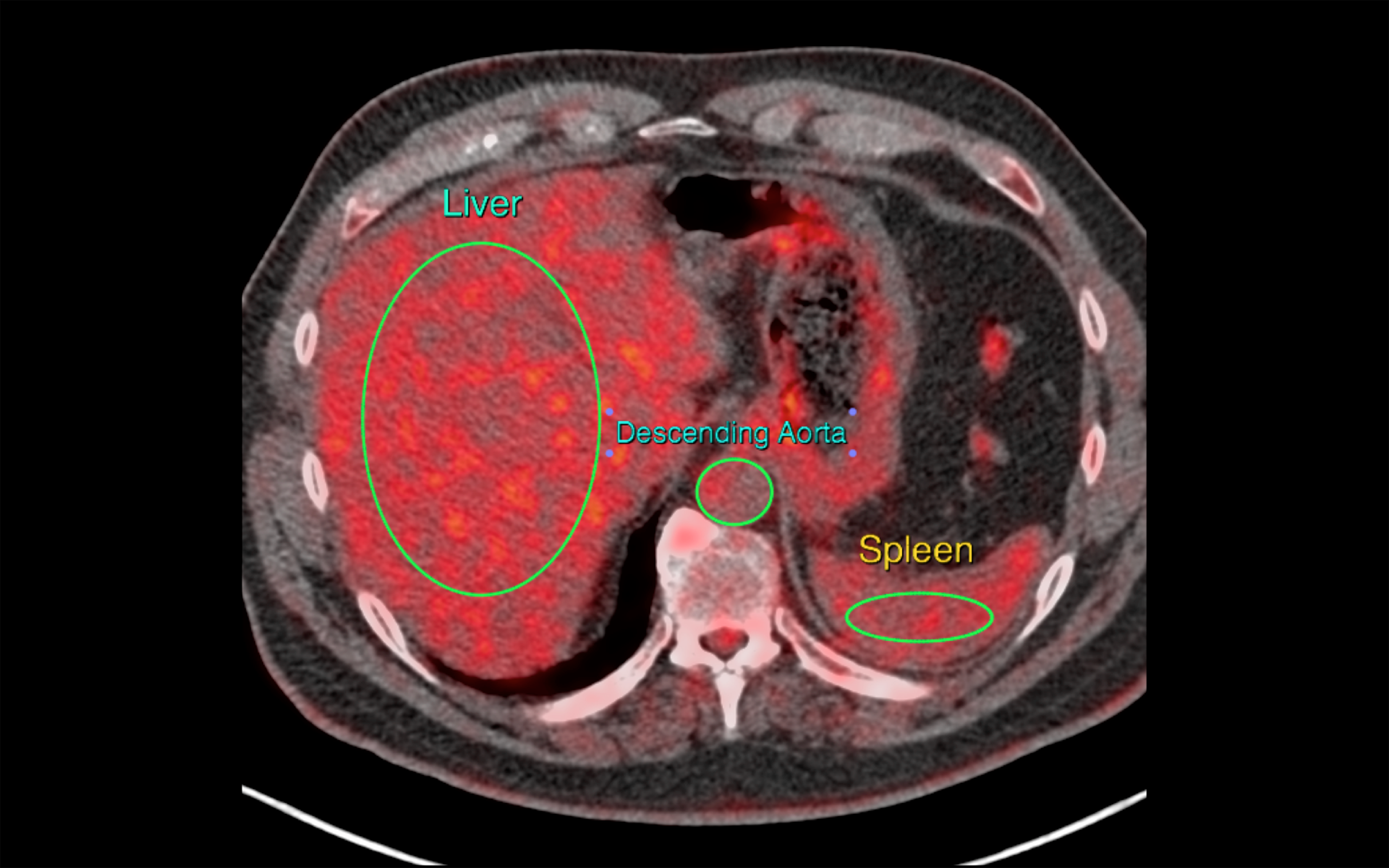

Supplement: S2 Fig — (TIF) [file pone.0187995.s002.tif]
