# Supplementary material for: Internal tissue references for 18Fluorodeoxyglucose vascular inflammation imaging: Implications for cardiovascular risk stratification and clinical trials
Source: PLoS One. 2017 Nov 13;12(11):e0187995. doi: 10.1371/journal.pone.0187995 (PMC5683610; doi:10.1371/journal.pone.0187995)
Supplement: S1 Table — (DOCX) [file pone.0187995.s004.docx]

**S1 Table. Measurement characteristics.**

|  | **Mean** | **Standard Deviation** | **COV%** |
| --- | --- | --- | --- |
| **Blood SUV** | 0.84 | 0.11 | 12.5 |
| **Liver SUV** | 1.51 | 0.20 | 13.2 |
| **Spleen SUV** | 1.32 | 0.21 | 15.7 |
| **Aorta SUV** | 1.99 | 0.29 | 14.5 |
| **TBR_Blood_** | 2.38 | 0.23 | 9.5 |
| **Blood Subtraction** | 1.65 | 0.32 | 19.1 |
| **TBR_Liver_** | 1.32 | 0.13 | 10.1 |
| **TBR_Spleen_** | 1.52 | 0.17 | 11.2 |


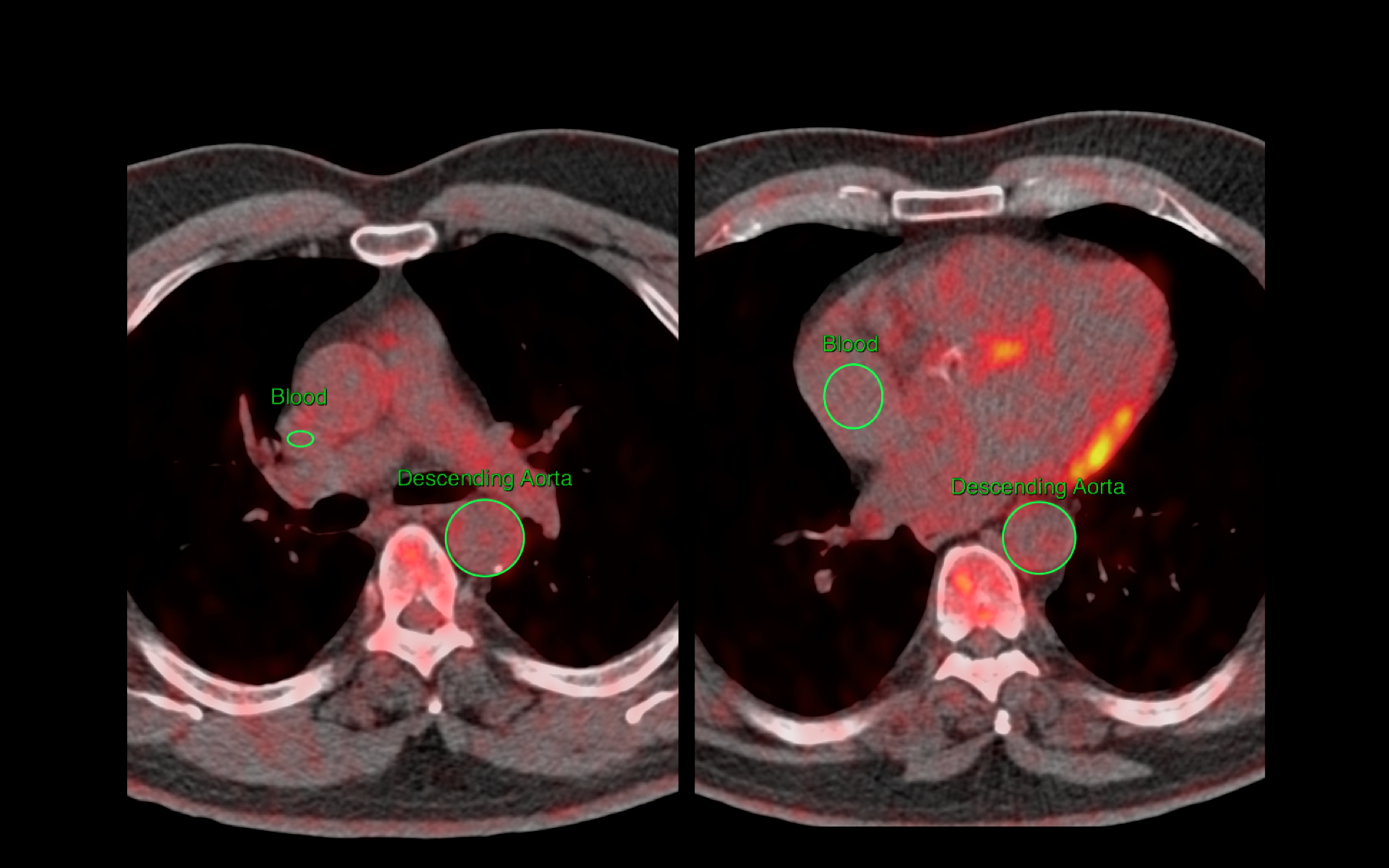
 **S1 Fig**. **Aorta wall and blood segmentation.** Two axial levels over the heart demonstrate whole-vessel wall segmentation of the descending aorta for sampling of maximum SUV. Regions of interest were placed for mean SUV measurement of blood activity over the large blood reservoirs across the caudocranial imaging plane (jugular, superior/inferior vena cava). Common structures that required avoidance for measurement of artery wall activity included the esophagus, myocardium, bone marrow, and bowel. For the measurement of blood activity, adjacent artery wall activity, myocardium, bone marrow, liver, and bowel activity required avoidance and/or minimization for overall averaging.


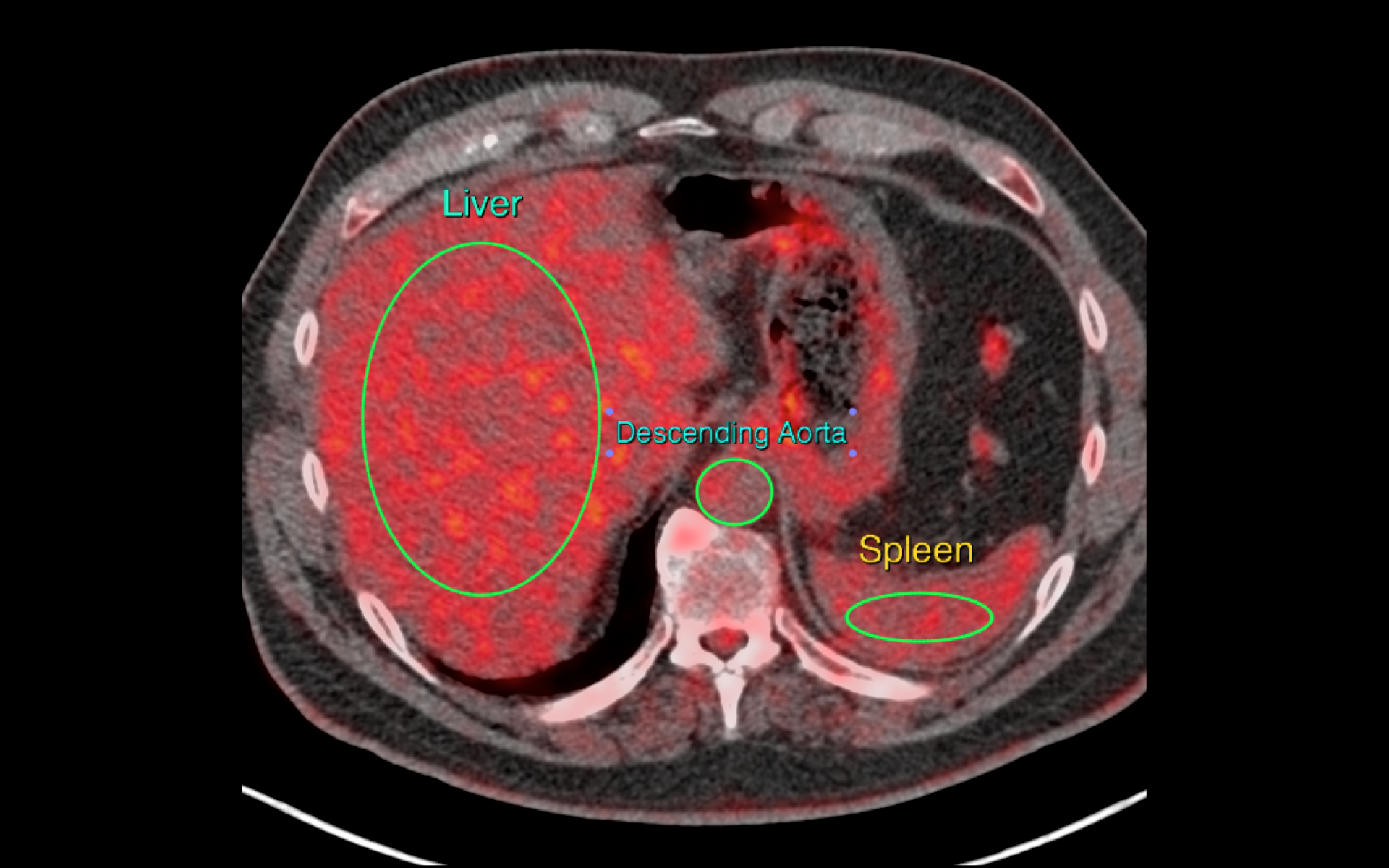


**S2 Fig. Segmentation of liver and spleen.** Large regions of interest were drawn over multiple slices over the liver and spleen with care to avoid non-organ activity. Regions of the organ that were inhomogenous at their margins along the diaphragm were avoided, as these areas were likely influenced by respiratory and/or attenuation artifact. The workflow included visual appreciation of both CT and PET images for the presence of the organs for segmentation. This image also shows a potential interference of adjacent esophageal activity on descending aorta segmentation at the gastric hiatus, which required a 3-dimensional check to assure that activity visually coincided with the expected intensity and geometry related to circumferential vessel wall activity.
